# High-Resolution 3D Magnetic Resonance Fingerprinting With a Graph Convolutional Network

**DOI:** 10.1109/TMI.2022.3216527

**Published:** 2023-03-02

**Authors:** Feng Cheng, Yilin Liu, Yong Chen, Pew-Thian Yap

**Affiliations:** Department of Computer Science, University of North Carolina, Chapel Hill, NC 27599 USA; Department of Computer Science, University of North Carolina, Chapel Hill, NC 27599 USA; Department of Radiology, Case Western Reserve University, Cleveland, OH 44106 USA; Department of Radiology and the Biomedical Research Imaging Center (BRIC), University of North Carolina, Chapel Hill, NC 27599 USA

**Keywords:** 3D magnetic resonance fingerprinting (MRF), graph convolution, GRAPPA, k-space interpolation

## Abstract

Magnetic resonance fingerprinting (MRF) is a novel quantitative imaging framework for rapid and simultaneous quantification of multiple tissue properties. 3D MRF allows higher through-plane resolution, but the acquisition process is slow when whole-brain coverage is needed. Existing methods for acceleration mainly rely on GRAPPA for k-space interpolation in the partition-encoding direction, limiting the acceleration factor to 2 or 3. In this work, we replace GRAPPA with a deep learning approach for accurate tissue quantification with greater acceleration. Specifically, a graph convolution network (GCN) is developed to cater to the non-Cartesian spiral sampling trajectories typical in MRF acquisition. The GCN maintains high quantification accuracy with up to 6-fold acceleration and allows 1 mm isotropic resolution whole-brain 3D MRF data to be acquired in 3 min and submillimeter 3D MRF (0.8 mm) in 5 min, greatly improving the feasibility of MRF in clinical settings.

## I. Introduction

Quantitative magnetic resonance imaging (MRI), such as T1 and T2 mapping, allows more definitive and objective tissue characterization for diagnosis, monitoring, and therapy assessment [1]. However, current quantitative MRI methods [2], [3] are slow and quantify only one tissue property per scan. Multiple MR imaging techniques have been recently developed to simultaneously quantify multiple tissue properties with high scan efficiency [4], [5]. Among them, magnetic resonance fingerprinting (MRF) is a relatively new quantitative imaging method that utilizes a highly accelerated data acquisition scheme for efficient simultaneous quantification of multiple tissue properties [6]. MRF characterizes each tissue type with a unique signal time course, called *fingerprint*, generated using pseudo-randomized imaging parameters. Multiple tissue properties are inferred simultaneously via template matching of an acquired signal time course with a dictionary of fingerprints corresponding to a wide range of tissue parameters. MRF has been extensively applied to various applications in brain, abdomen, heart, and breast imaging [7], [8], [9].

For applications where contiguous volumetric coverage is crucial, several studies [8], [10] have extended MRF acquisition from 2D to 3D using stack-of-spirals for improving coverage, signal-to-noise ratio, and spatial resolution. However, the acquisition time of 3D MRF for high-resolution whole-brain coverage is prohibitively long for clinical settings. Liao et al. [11] employ a hybrid GRAPPA and sliding window approach for acceleration along the partition-encoding direction in k-space. Chen et al. [12] combine non-Cartesian parallel imaging with deep learning, achieving 2-fold k-space and 4-fold temporal acceleration, allowing 1 mm isotropic resolution and whole-brain coverage 3D MRF data to be acquired in 7 min. Further acceleration is desirable for clinical applications to alleviate patient discomfort, improve scan efficiency and throughput, and reduce motion artifacts. Moreover, non-Cartesian GRAPPA reconstruction in k-space is time-consuming, prohibiting real-time display of tissue parameter maps.

Recent studies have shown that deep learning (DL) can mitigate aliasing artifacts resulting from undersampling in parallel imaging. Lee et al. [13] proposed a deep residual learning method to accelerate MRI in the image space using magnitude and phase images. Eo et al. [14] proposed KIKI-net to accelerate MRI using information in both k-space and image space. Akcakaya et al. [15] proposed a deep learning method, called RAKI, for parallel imaging in k-space. Similar to GRAPPA, RAKI is trained on calibration data acquired at the k-space center and applied to infer missing k-space lines. However, most of these DL methods are designed for Cartesian MRI, while many applications such as MRF are reliant on non-Cartesian sampling.

Deep learning methods have recently been developed for multiple aspects of MRF, including dictionary generation [16], acquisition acceleration [12], [17], reconstruction [18], [19], and others [20], [21], [22]. In this paper, we introduce a graph convolutional network (GCN), which caters to the non-Cartesian nature of the sampling trajectory typical in 3D MRF, for replacing spiral GRAPPA for higher acceleration along the partition-encoding direction with the goal of significantly shortening acquisition time. Combined with deep learning based tissue quantification [12], our approach allows 3D whole-brain MRF data to be acquired at 1 mm isotropic resolution for simultaneous T1 and T2 mapping in less than 4 min. Performance evaluation under multiple acceleration factors demonstrates that our approach maintains high quantification accuracy even with an acceleration factor of 4. Extending the conference version of this work [23],

- We increased the acceleration factor to 6, further reducing the acquisition time to ~3min for whole-brain coverage at 1 mm isotropic resolution.
- We extended our method for 0.8 mm isotropic resolution with an acquisition time of ~5 min.
- We evaluated our method on prospectively undersampled data with whole-brain coverage at 0.8 mm resolution.
- We performed extensive comparison of the our GCN with GRAPPA and a Cartesian CNN.

## II. Materials and Methods

### A. Data Acquisition

Two sets of 3D MRF data were acquired at two different spatial resolutions (1 × 1×1 mm^3^ and 0.8 × 0.8×0.8 mm^3^) using a Siemens 3T scanner with a 32-channel receive coil. Written consent was obtained from each subject before the MRI experiment. The pulse sequence was based on a stack-of-spirals trajectory and steady-state free precession (SSFP) readout [8]. Similar to 2D MRF, pseudorandomized flip angles and a highly-undersampled spiral readout was employed. For each MRF time frame (a 3D volume highly-undersampled in k-space), the same spiral arm was acquired across all the partitions (in-plane acceleration factor: 48) and golden-angle spirals were applied across MRF time frames [8]. Multiple inversion-recovery (inversion time: 20 – 400ms) and T2-preparation (effective echo time: 50 or 90 ms) modules were employed to improve sensitivity to both T1 and T2 values [24].

Acquisition was carried out sequentially along the partition-encoding direction with the same acquisition pattern (flip angles, spiral sampling, etc.) for each partition. A constant wait time of 2 s was included at the end of the acquisition of each partition for longitudinal recovery. Each partition was acquired across a total of 768 MRF time frames, a TR each, in 16 segments with 48 TRs per segment. A variable wait time between 190 ms and 440 ms was applied at the end of each segment for variable longitudinal recovery [8]. The acquisition time per partition was 15 s for 1 mm isotropic resolution (5.9 ms per spiral readout) and 18 s for 0.8 mm resolution (8.8 ms per spiral readout).

Imaging parameters specific to 1 mm acquisition include matrix size: 256 × 256; field of view (FOV): 25 × 25cm^2^; TR: 9.2 ms; TE: 1.3 ms; and flip angles: 5–12 degrees. 3D MRF data were fully acquired along the partition-encoding direction and retrospective data undersampling was utilized to generate data for various acceleration factors. A total of 144 sagittal partitions were acquired with 6/8 partial Fourier sampling. The total acquisition time for one full 3D MRF scan was about 30 min. For each subject, 12 auto-calibration signal (ACS) slices at the center k-space were acquired for network training and conventional GRAPPA reconstruction. Data was acquired from 6 subjects (male/female: 3/3; age: 34 ± 10).

Imaging parameters specific to 0.8 mm acquisition include matrix size: 320 × 320; FOV: 25 × 25cm^2^; TR: 12.6 ms; TE: 1.3 ms; and flip angles: 5–12 degrees. A total of 176 sagittal partitions were acquired with 2× interleaved k-space undersampling along the partition-encoding direction (partial Fourier: 6/8; ACS slices: 16; total acquisition time per subject: ~20 min). Data was acquired from 5 subjects (male/female: 3/2; age: 27 ± 6). The data was retrospectively undersampled for numerical evaluation. An additional prospectively undersampled scan with 0.8 mm resolution and whole-brain coverage (14 cm sagittal) was acquired for further evaluation of method feasibility.

We denote the acquired MRF data as $X\in C^{T\times P\times Q\times C}$, where T=768, P=144, or 176, Q=2452 or 3300, and C=32 denote the numbers of time frames, partitions, spiral readout points, and coil channels, respectively, for the two spatial resolutions.

### B. Approach

**Fig. 1.**
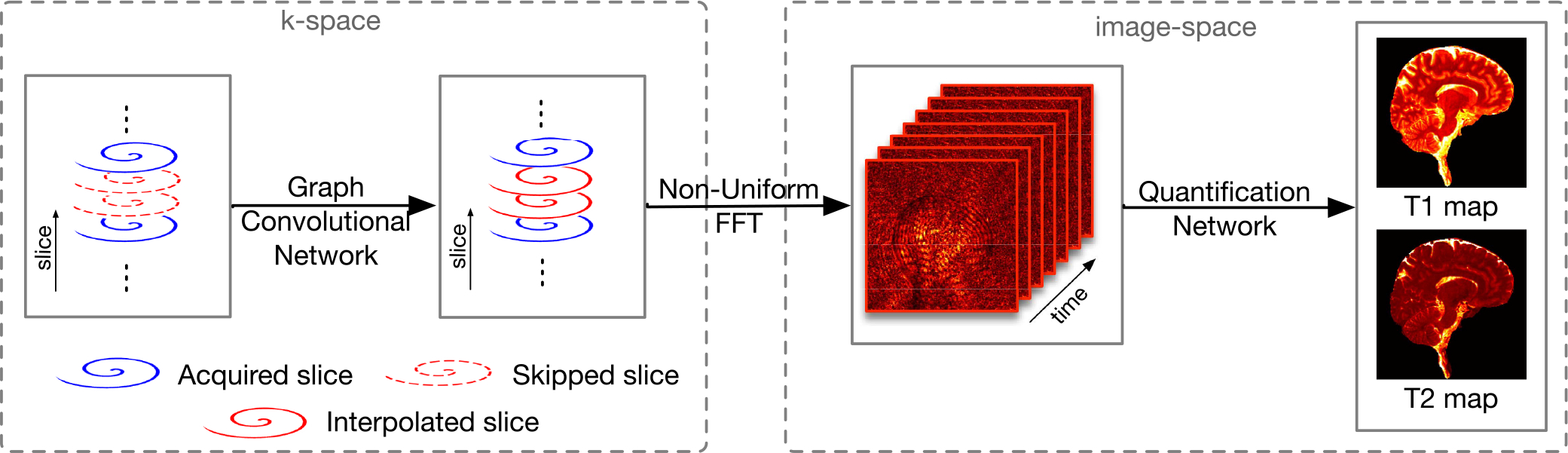
Our method involves (i) deep learning reconstruction of k-space data, (ii) k-space to image space transformation via non-uniform fast Fourier transform (NUFFT), and (iii) deep learning T1 and T2 quantification [17].

Our method, summarized in Fig. 1, involves (i) deep learning k-space data interpolation along the partition-encoding direction, (ii) k-space to image space transformation via non-uniform fast Fourier transform (NUFFT), and (iii) deep learning T1 and T2 quantification [17]. We will first review spiral GRAPPA and a Cartesian CNN, and then flesh out our method.

#### 1) Spiral GRAPPA:

**Fig. 2.**
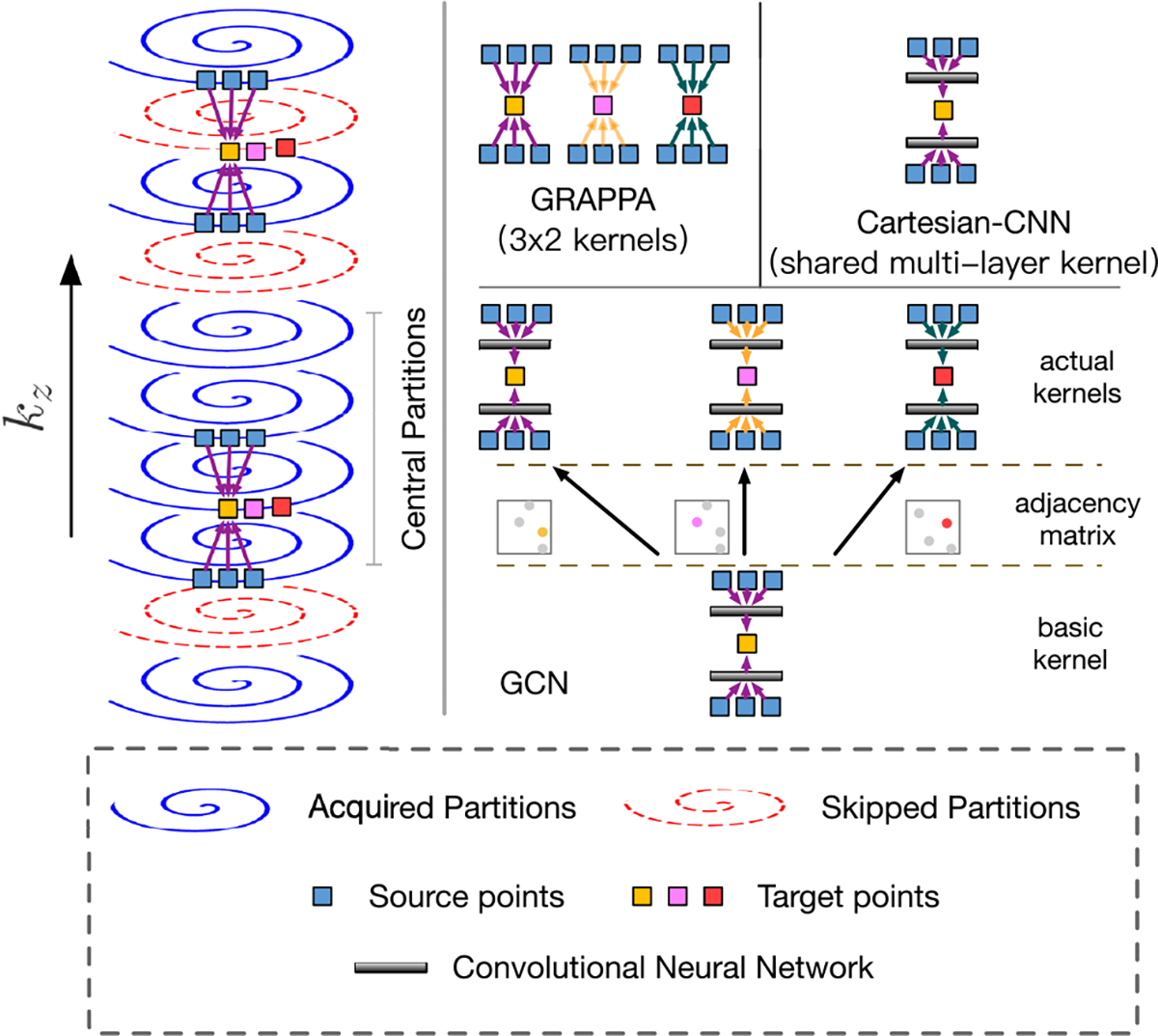
Interpolation of spiral k-space data. Left: Kernels are learned from the central ACS partitions and applied to other partitions at the same location in the spiral-readout direction. Cartesian CNN treats the spiral points as equally spaced. The GCN explicitly considers the non-uniform locations of the spiral points while learning network weights.

The non-Cartesian spiral GRAPPA was first proposed by Seiberlich et al. [25] for cardiac imaging. It was extended by Chen et al. [12] to accelerate 3D MRF along the partition-encoding direction. As shown in Fig. 2, each point on the spiral arm in a missing partition is predicted with a 2 × 3 GRAPPA kernel determined based on ACS data acquired in central partitions. For the *c*-th coil channel, the *i*-th k-space point of the *m*-th partition is estimated as

(1) $$S_{c}(m,i)=\sum_{c^{\prime }=1}^{N_{\text{coil}\;}}\sum_{m^{\prime }\in \{0,R\}}\sum_{i^{\prime }=i-v}^{i+v}g\left(m^{\prime },i^{\prime }|m,i,c\right)S_{c^{\prime }}\left(m^{\prime },i^{\prime }\right),$$

where m=1,…,R−1,R is the acceleration rate, 0 and R are the indices of two acquired partitions, and $g\left(m^{\prime },i^{\prime }|m,i,c\right)$ is the GRAPPA kernel. The GRAPPA kernel is of size 2×(2v+1) and is used to convolve over 2 partitions, each with 2v+1 points. Unlike in Cartesian MRI, points on a spiral arm are not spaced equally. Therefore, the GRAPPA kernel for each spiral point neighborhood varies with location.

Spiral GRAPPA can be written in matrix form as

(2) H=SW

in which $S\in C^{1\times (Q\times C\times 2)}$ is the nearest two acquired spirals, $H\in C^{1\times (Q\times C\times (R-1))}$ is the prediction of the missing spirals and $W\in C^{\left(Q\times C\times 2)\times (Q\times C\times (R-1)\right)}$ denotes the kernel weights that are estimated via least squares. Each column of W is a GRAPPA kernel g(⋅) padded with zeros.

Despite reasonably effective for 3D MRF [12], spiral GRAPPA has the following limitations:

- GRAPPA kernels are linear in nature as indicated by (2), and thus may limit approximation ability.
- Calibration data per kernel is much fewer than in Cartesian MRI, potentially leading to overfitting.
- The kernels (columns of W in (2)) are learned independently without harnessing their interrelationships.

#### 2) Cartesian CNN:

By assuming equally-spaced spiral points and “straightening” the spirals, a Cartesian CNN can be applied for k-space interpolation in MRF. Unlike GRAPPA, the Cartesian CNN estimates the missing spiral points via non-linear activation functions with kernels shared by all spiral neighborhoods. This is realized via multiple convolutional layers:

(3) $$H^{\left(l+1\right)}=\sigma (conv(H^{\left(l\right)};W^{\left(l\right)})),$$

where $H^{\left(l+1\right)}\in C^{Q\times C_{l+1}}$ is the output of the l-th layer, $W^{\left(l\right)}\in C^{(2v+1)\times C_{l}\times C_{l+1}}$ is a learnable kernel shared by all readout points, and σ(⋅) is the ReLU non-linear activation function. Input $H^{\left(0\right)}$ consists of two acquired spirals concatenated across coil channels. After N layers, $H^{\left(N\right)}$ denotes the prediction of a missing spiral. The kernels of the CNN are expected to be significantly more expressive than GRAPPA.

**Fig. 3.**
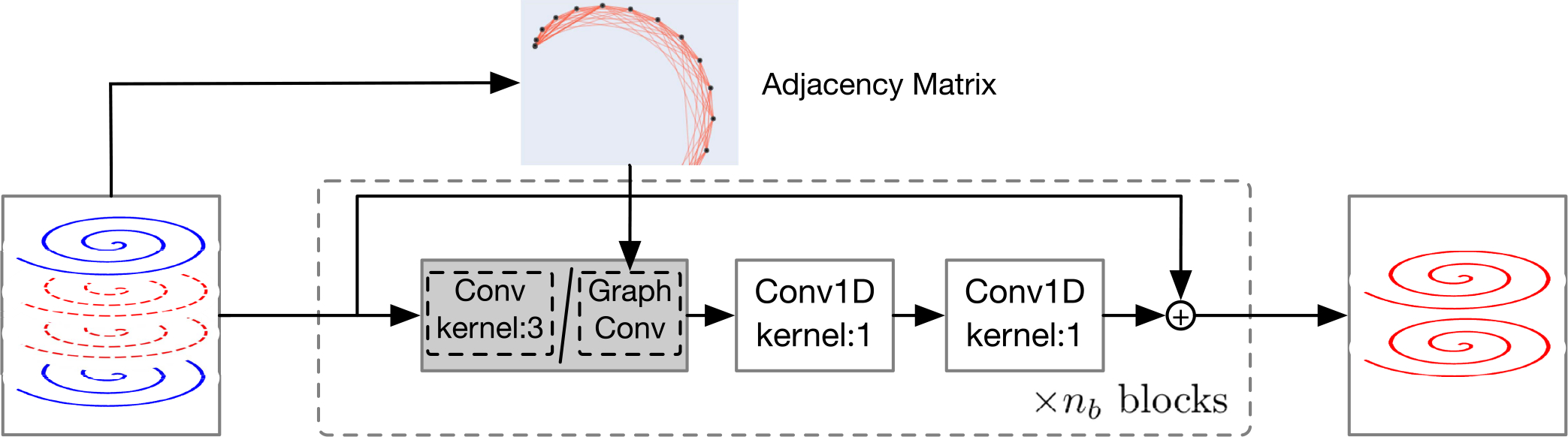
The Cartesian CNN (kernel size 3) and the graph convolutional network (GCN). In this example, the acceleration factor is *R* = 3.

As shown in Fig. 3, the network consists of $N_{\text{block}}$ blocks, blocks, each formed with one 1D convolution layer with kernel size 3 followed by two 1D convolution layers with kernel size 1. Residual connections [26] are added in each block to ease network training. All the convolutional layers are ReLU activated, except the last layer. Within each block, the first convolution layer with kernel size 3 aggregates information from neighbors similar to GRAPPA, except that neighboring points are assumed equally spaced. The two subsequent convolutions with kernel size 1 map the data to a high-dimensional space to learn inter-point relationships. Essentially, the Cartesian CNN reformats the spirals as straight lines and disregards the non-uniformity of the spiral points. Non-linear reconstruction is learned using all the points on the spirals.

#### 3) Graph Convolutional Network (GCN):

To explicitly account for the non-uniform distribution of the spiral points, we represent each spiral trajectory as a graph and employ graph convolutions throughout the network in Fig. 3, as inspired by [27]. Unlike GRAPPA, the GCN employs kernels that are adaptive to different point neighborhoods (Fig. 2). In contrast to the Cartesian CNN, the GCN takes into account not only the features of the neighboring points but also their positions relative to the missing point, inherently catering to varying point-neighborhood configurations. We will demonstrate with empirical results that the number of network parameters of the Cartesian CNN needs to be increased significantly to achieve performance similar to the GCN. To ensure fair comparison, the GCN is designed to have a network architecture similar to the Cartesian CNN, with only the convolutional layers replaced by graph convolutional layers (Fig. 3).

The relationships between k-space points of a partition are encoded in an adjacency matrix A of a graph 𝒢=(𝒱,ℰ), with vertices 𝒱 representing the k-space points and edges ℰ representing the relationships between the points in k-space. The adjacency matrix A is defined as

(4) $$A_{i,j}=\exp\left(-\frac{{\left|v_{i}\;-\;v_{j}\right|}^{2}}{{\overset{‾}{d}}^{2}}\right),$$

where $v_{i}$ and $v_{j}$ are the 2D coordinates of a pair of k-space points, and $\overset{‾}{d}$ is the mean distance of all pairs of points on a spiral arm. Only K nearest neighbors for each vertex, including the vertex itself, is retained by keeping the K largest values in each row of A and setting the others to 0. Graph convolution is performed with kernel size K as [27]

(5) $$H^{\left(l+1\right)}=\sigma ({\overset{ˆ}{D}}^{-1/2}\overset{ˆ}{A}{\overset{ˆ}{D}}^{1/2}H^{\left(l\right)}W^{\left(l\right)}),$$

where $\overset{ˆ}{A}=A+I$ and D is a diagonal degree matrix with ${\overset{ˆ}{D}}_{ii}=\sum_{j}A_{ij},W^{\left(l\right)}$ is a matrix of kernel weights, $H^{\left(l\right)}$ is the matrix of activations in the l-th layer, and σ(⋅)=max(0,⋅) is the ReLU activation function.

#### 4) Implementation:

For both 3D MRF datasets, one subject was used for testing and the rest were used for training. The GCN was trained using 12 (for 1 mm resolution) or 16 (for 0.8 mm resolution) ACS central partitions with a stride of 1. During inference, the GCN was applied to predict all skipped partitions. Each training sample consists of two adjacent acquired partitions as input $\left(C^{2\times Q\times C}\right)$ and R−1 skipped partitions as target $\left(C^{(R-1)\times Q\times C}\right)$. The real and imaginary parts were stacked across coil channels. The three convolutional layers in each block had 512, 1024, and 1024 filters. The mean squared error between the GCN output and the sampled k-space points in the ACS data was employed as the loss function. Optimization was performed using ADAM with an initial learning rate $5\times 10^{-4}$, decayed by a factor of 99% at the end of each epoch. The batch size was 1. NUFFT was applied to reconstruct the 3D MRF data from k-space. Similar to [17], a spatially-constrained U-Net was applied, in place of standard template matching, to predict T1 and T2 values (Fig. 1). The network takes as input the reconstructed MRF time frames $\left(C^{T\times M\times M}\right)$ and outputs the tissue property maps ($R^{M\times M}$ for each map).

## III. Experiments

Evaluation was performed for the 1 mm MRF dataset for multiple acceleration factors (*R* = 2, 3, 4, 5, 6) in the partition-encoding direction. Taking into account the 12 ACS partitions, the effective acceleration factors were 1.8, 2.6, 3.2, 3.8 and 4.2, respectively. Evaluation for the 0.8 mm dataset was focused on acceleration factor 4. Further evaluation was performed with the prospective scan described in Section II-A. For both datasets, we used only the first 192 of the 768 time frames for an acceleration of factor 4 in the temporal dimension, similar to [12]. Considering the 2 s wait time between partitions, the effective acceleration factor was ~3 for both resolutions. The ground-truth T1 and T2 maps for the 1 mm dataset were generated using all time frames, fully-sampled in the partition-encoding direction. For the 0.8 mm dataset, the ground-truth maps were obtained using GRAPPA followed by template matching [12].

### A. Comparison With State-of-the-Art (SOTA) Methods

We compared the Cartesian CNN and the GCN with two SOTA methods—Spiral GRAPPA [12] and RAKI [15]—in terms of k-space reconstruction and tissue property quantification. RAKI is constructed with 3 convolutional layers and operates on straight lines reformatted from the spirals.

#### 1) k-Space Reconstruction:

Similar to [15], k-space reconstruction accuracy was evaluated using the normalized mean square error (NMSE). The results for 3D MRF with 1 mm resolution and three different acceleration factors (2, 3, and 4) are summarized in Table I, giving the following conclusions:

- The one-block GCN-1B (*N*_block_ = 1) outperforms the SOTA methods.
- The three-block GCN-3B (*N*_block_ = 3) yields the lowest NMSE.
- Compared with the Cartesian CNN, the GCN-3B performs better and faster with less number of parameters.
- The deep learning methods are over an order of magnitude faster than GRAPPA.

Results for ablation studies comparing the GCN-1B and the GCN-3B are reported in Section III-B.2.

#### 2) Tissue Property Quantification:

**Fig. 4.**
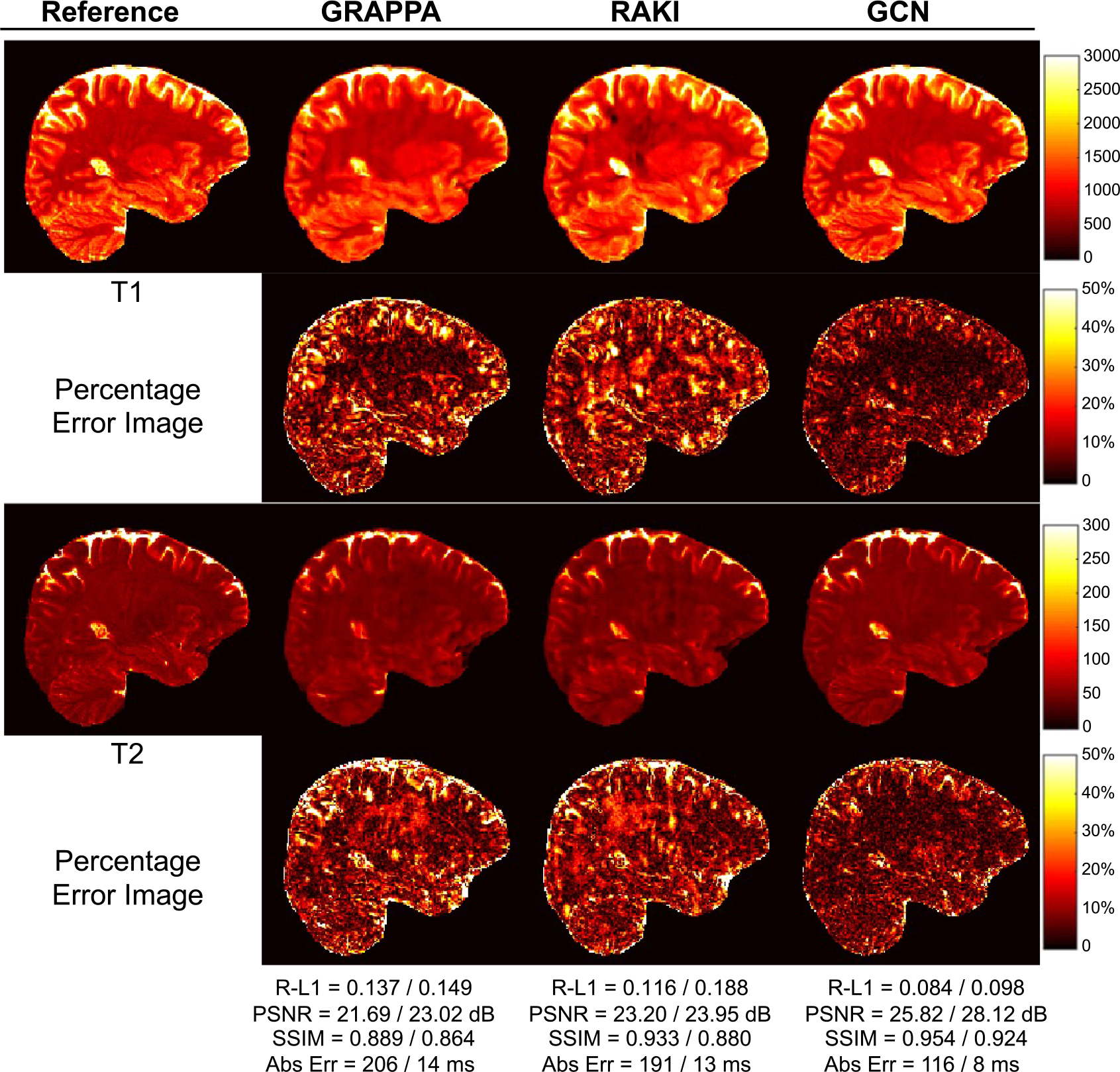
Representative T1 and T2 maps for *R* = 4 with the corresponding T1/T2 numerical results shown at the bottom (1 mm resolution).

**Fig. 5.**
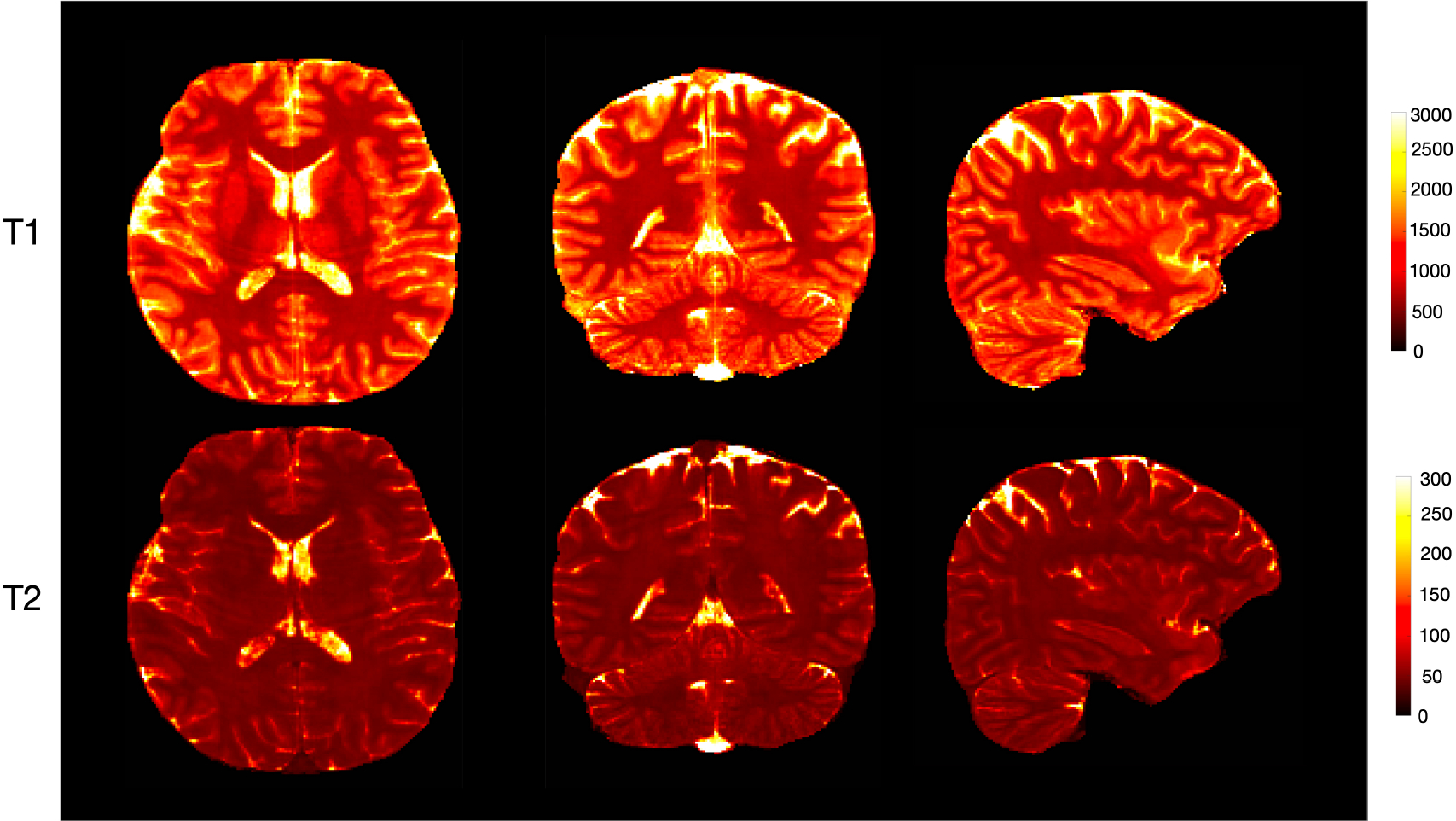
Whole-brain T1 and T2 maps generated using the GCN with *R* = 4 in axial, coronal, and sagittal views. The acquisition time for 144 sagittal slices is less than 4 min (1 mm resolution).

Following [28], [29], performance in tissue property quantification was evaluated using Relative-L1, PSNR, SSIM, and absolute errors in milliseconds. As shown in Table II, both the Cartesian CNN and the GCN ourperform spiral GRAPPA and RAKI for all metrics and *R* = 2, 3, 4. The GCN performs comparably with the Cartesian CNN, but faster with 40% less parameters (see Table I). Figure 4 shows representative tissue maps for *R* = 4. Compared with the SOTA methods, the T1 and T2 maps obtained with the GCN exhibit less artifacts with smaller errors, better contrasts, and clearer structural details. The errors vary across tissue types. For example, for the GCN results obtained with *R* = 4, the T1 R-L1 errors for WM, GM, and CSF are 0.051, 0.090, and 0.135, respectively, whereas the T2 R-L1 errors for WM, GM, and CSF are 0.062, 0.092, and 0.214, respectively. Figure 5 shows the T1 and T2 maps in three different views for *R* = 4. With the GCN, the acquisition time for whole brain coverage is reduced to 5 min with *R* = 3 and 4 min with *R* = 4.

**Fig. 6.**
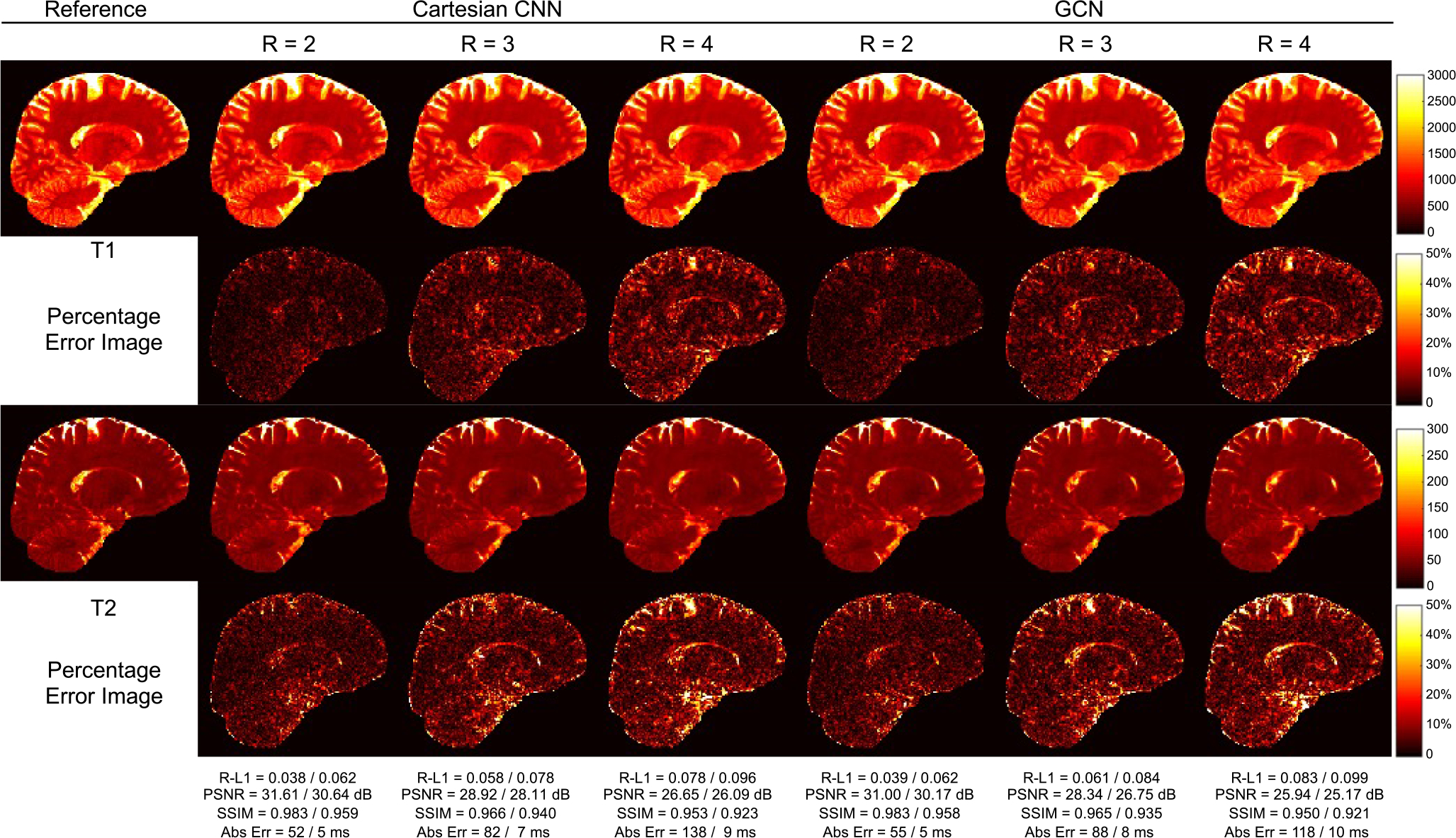
Comparison of T1 and T2 maps between the Cartesian CNN and the GCN (1 mm resolution).

**Fig. 7.**
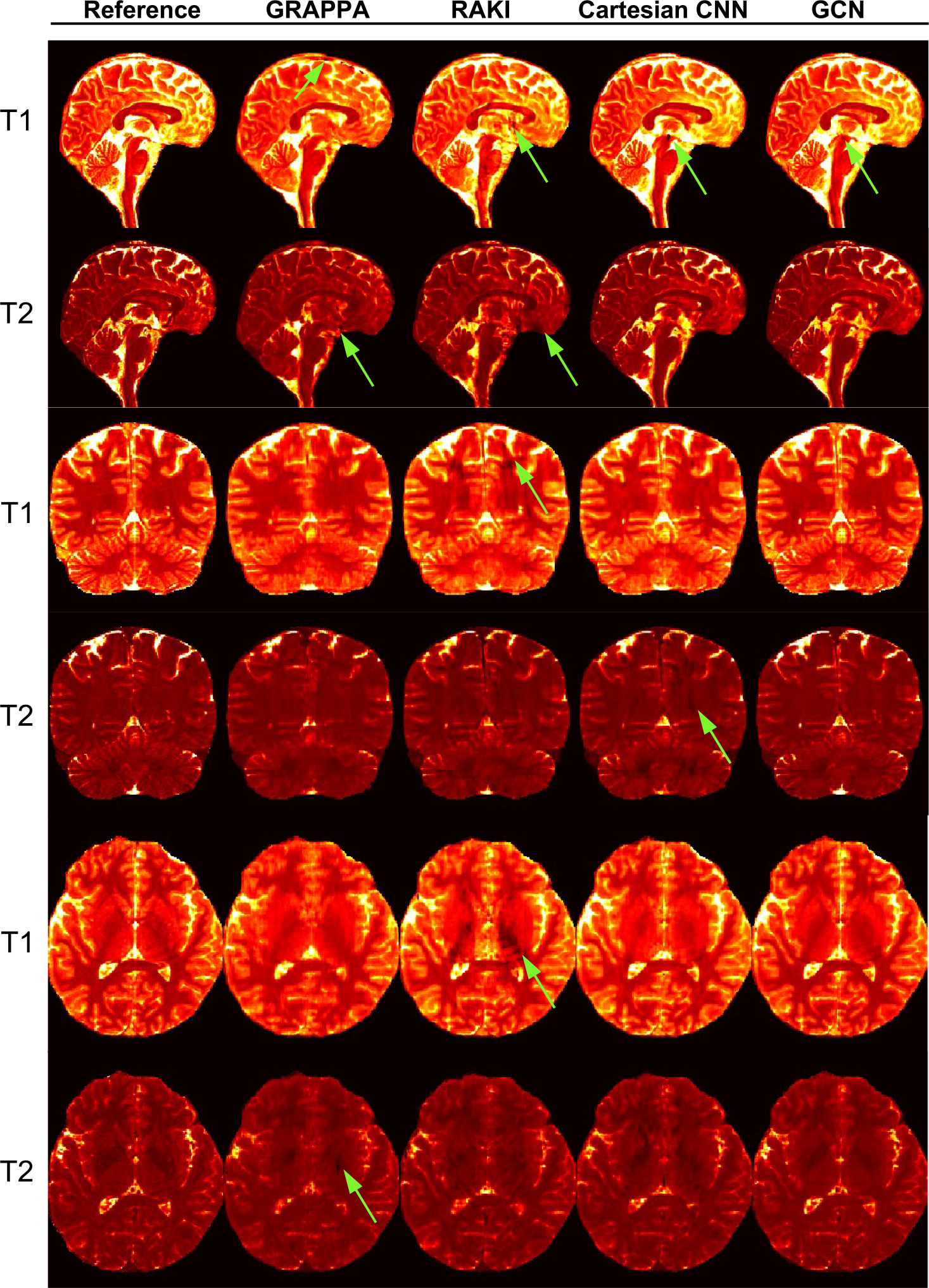
T1 and T2 maps in sagittal, coronal, and axial views generated by various methods for *R* = 4 (1 mm resolution). Apparent artifacts are marked by green arrows.

Figure 6 shows representative T1 and T2 maps obtained using the Cartesian CNN and the GCN with three different acceleration factors (2, 3, and 4). Although they show similar performance, apparent artifacts in the form of dark holes can be observed for GRAPPA, RAKI, and the Cartesian CNN (Figure 7). Cartesian deep learning methods, i.e., RAKI and the Cartesian CNN, yield in overall better quantification results than GRAPPA, but are more susceptible to artifacts. The GCN is the most effective and yields the best quantification results without apparent artifacts.

### B. Ablation Study

We performed ablation studies to investigate factors that influence the performance of the GCN, using the 1 mm dataset.

#### 1) Kernel Size:

The kernel size *K* of the GCN controls the receptive field and determines the number of points each point can draw information from. The best performance is given by *K* = 5 (Table III).

#### 2) Number of Blocks:

Table IV shows that *N*_block_ = 3 or *N*_block_ = 5 gives the best performance. Insufficient blocks will limit network capacity. Too many blocks will cause overfitting to limited samples.

### C. Higher Acceleration

**Fig. 8.**
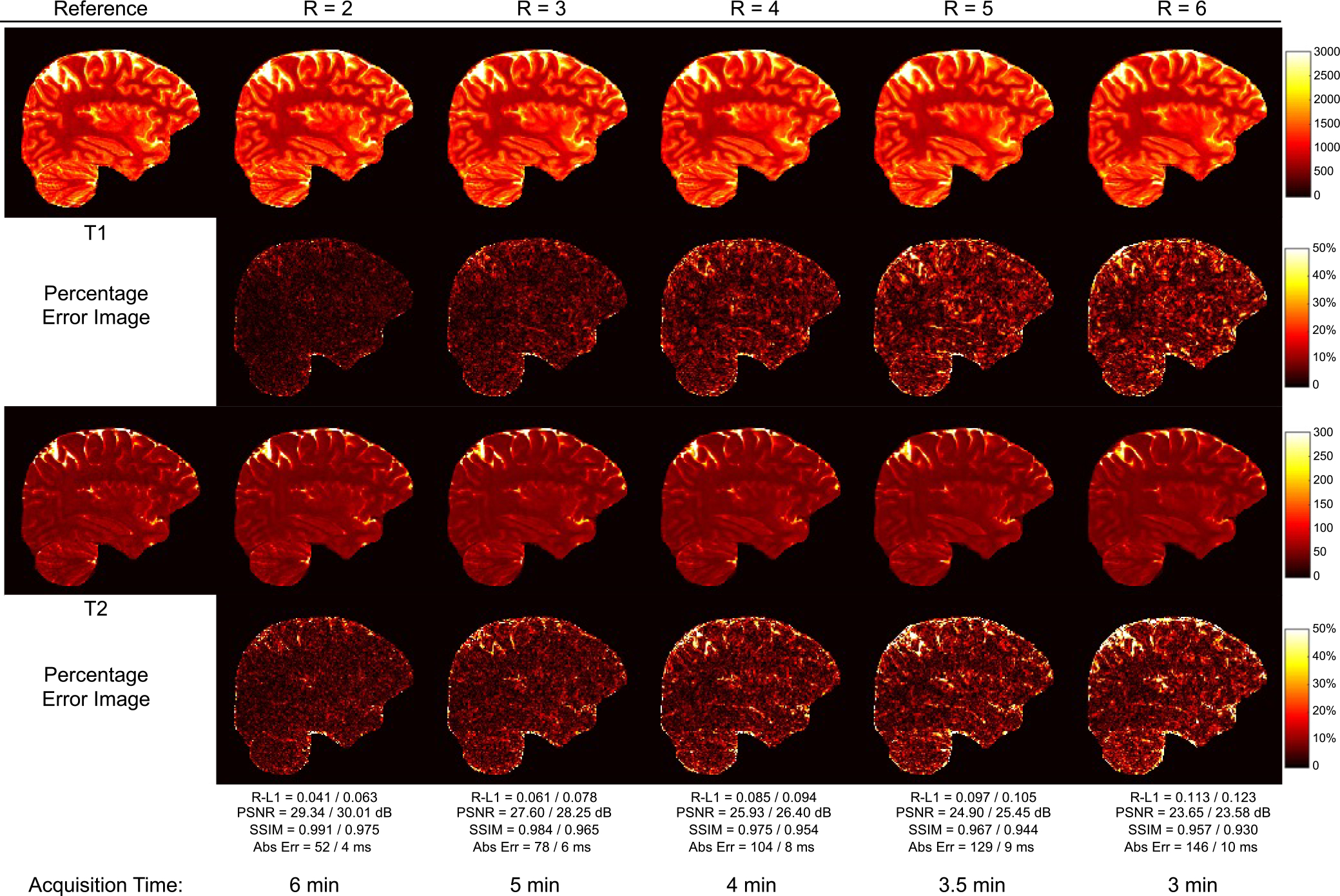
T1 and T2 maps generated by the GCN for various acceleration factors (1 mm resolution).

We further explored higher acceleration of factor 6 along the partition-encoding direction. Figure 8 shows representative T1 and T2 maps reconstructed with the GCN from retrospectively undersampled data. Similar to previous observations, no artifacts are observable even at high acceleration of factor 6. A total of 24× acceleration is achieved when combined with 4× acceleration along the temporal dimension, reducing the total scan time to approximately 3 min for 1 mm 3D MRF with whole-brain coverage. Relative-L1, PSNR, and SSIM values for *R* = 6 are summarized in Table V. The GCN performs similar to or better than the Cartesian CNN at 6× acceleration.

### C. Submillimeter 3D MRF

**Fig. 9.**
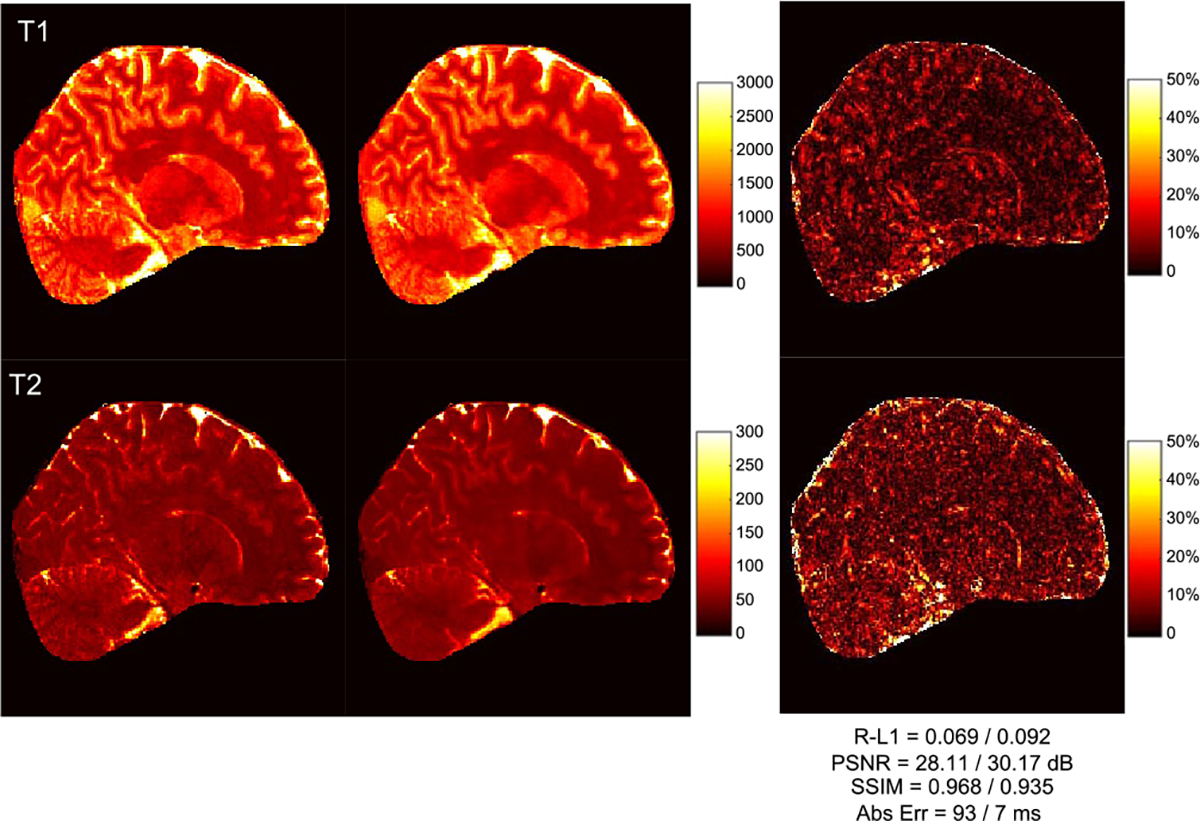
Tissue property maps reconstructed from prospectively undersampled MRF data with 0.8mm isotropic resolution and *R* = 4. The data was acquired within 5 min.

**Fig. 10.**
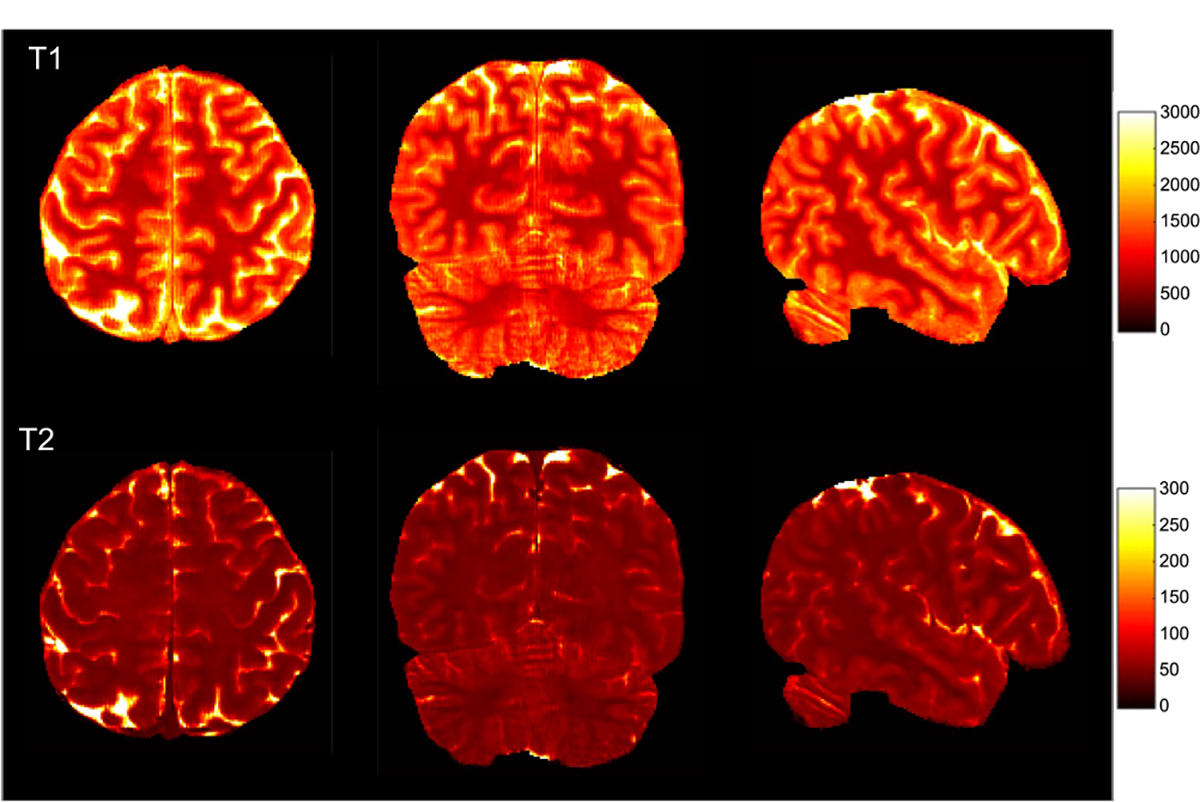
Whole-brain T1 and T2 maps reconstructed from the 0.8 mm MRF data with 4× acceleration along the partition-encoding direction (5 min acquisition time).

We applied the GCN for 0.8 mm 3D MRF. Figure 9 shows representative T1 and T2 maps obtained with 4× retrospective undersampling along the partition-encoding direction. Figure 10 shows in three different views the T1 and T2 maps obtained from the prospectively undersampled data, which was acquired in 5 min. Similar to 1 mm 3D MRF, high-quality submillimeter quantitative maps can be reconstructed rapidly with the GCN.

## IV. Discussion

In this work, we employed a GCN to accelerate high-resolution 3D MRF along the partition-encoding direction, achieving acceleration factors up to 6. Our method substantially reduced both acquisition time and reconstruction time for high-resolution quantitative brain MRI.

In addition to comparing with GRAPPA, a conventional method, and RAKI, a state-of-the-art deep learning method, we demonstrated the efficacy of the GCN over the Cartesian CNN. While comparable to the GCN in terms of reconstruction accuracy at various acceleration factors, the Cartesian CNN requires a significantly larger number of parameters to sufficiently capture the data characteristics of 3D MRF.

Compared with conventional GRAPPA and the Cartesian CNN, the GCN yields improved map quality for both T1 and T2 quantification. With retrospective undersampling, some minor imaging artifacts were noted in the reformatted axial images (Figure 5). Similar artifacts can be found in the ground truth maps of a few subjects and are likely due to motion artifacts caused by long acquisition times. The focus of our approach is not on artifact removal. When applied to prospectively undersampled data that can be acquired rapidly in less than 5 min, our method predicted tissue property maps with no visible artifacts.

Our method has not been tested on pathological data. However, as GRAPPA generalizes well to pathology and both GRAPPA and our method learn k-space interpolation based on the ACS data, we expect our method to be generalizable to pathology like GRAPPA.

Although our method is focused on accelerating high-resolution 3D MRF, it is expected to be applicable to 3D MRF with lower spatial resolutions given the higher signal-to-noise ratio.

To demonstrate the utility of the GCN in accelerating 3D MRF, we opted for the most common choice of adjacency matrix based on Gaussian-weighted Euclidean distance of data points. There are multiple GCN variants with different adjacency formulations. Exploration of more customized adjacency matrices for MRF can be investigated in the future.

Finally, the performance of our method was evaluated based on the reconstructed T1 and T2 maps. More in-depth evaluation can be performed based on the predicted k-space data, for example, by studying the manifold of the predictions compared to the Bloch model based on T1 and T2 maps [30]. This could potentially lead to the development of manifold constraints for improved k-space reconstruction.

## V. Conclusion

In this work, we have introduced a graph convolutional network (GCN) to replace GRAPPA for greater acceleration along the partition-encoding direction for high-resolution 3D MRF. Combined with deep learning tissue quantification [17], our method reduces the acquisition time to 3 min for 1 mm isotropic resolution and 5 min for 0.8 mm isotropic resolution. Additionally, the reconstruction time can be substantially reduced by more than an order of magnitude over GRAPPA for whole-brain 3D MRF.

## Figures and Tables

**TABLE I:** Performance in K-Space Reconstruction

	NMSE			Time (minutes)			Params (millions)
	*R* = 2	*R* = 3	*R* = 4	*R* = 2	*R* = 3	*R* = 4	

GRAPPA [12]	0.930	1.420	1.560	97.6	132.0	301.5	1.97
RAKI [15]	0.401	0.568	0.731	7.2	5.3	4.8	1.36
Cartesian CNN	0.392	0.501	0.665	6.8	5.7	5.4	5.73
GCN-1B	0.384	0.531	0.705	**3.6**	**3.3**	**3.1**	**1.31**
GCN-3B	**0.377**	**0.500**	**0.663**	6.1	5.1	4.7	3.41

**TABLE II:** Performance in Tissue Quantification. The GCN and the Cartesian CNN are Both Implemented With 3 Blocks

		Relative-Ll			SSIM			PSNR		
		*R* = 2	*R* = 3	*R* = 4	*R* = 2	*R* = 3	*R* = 4	*R* = 2	*R* = 3	*R* = 4

T1	GRAPPA	0.077	0.107	0.152	0.978	0.962	0.929	26.26	24.09	21.14
	RAKI	0.069	0.097	0.118	0.984	0.971	0.958	26.16	24.01	23.15
	Cartesian CNN	**0.047**	**0.065**	**0.084**	**0.990**	**0.982**	**0.973**	**29.41**	**27.58**	**25.86**
	GCN	0.048	0.069	0.087	**0.990**	0.981	0.972	29.09	27.02	25.56

T2	GRAPPA	0.101	0.132	0.173	0.959	0.938	0.906	26.59	24.09	21.02
	RAKI	0.093	0.118	0.166	0.965	0.948	0.925	26.72	24.32	21.98
	Cartesian CNN	0.080	**0.094**	**0.113**	**0.975**	**0.965**	**0.954**	**29.72**	**28.23**	**26.65**
	GCN	**0.078**	0.097	0.115	**0.975**	0.963	0.953	29.44	27.55	26.25

**TABLE III:** K-Space Interpolation Errors for Different Kernel Sizes, K’s, With *N*_block_ = 3

	NMSE				
	*K* = 1	*K* = 3	*K* = 5	*K* = 7	*K* = 9

*R* = 2	0.397	0.379	**0.377**	0.395	0.432
*R* = 3	0.530	0.514	**0.500**	0.529	0.558
*R* = 4	0.686	0.671	**0.663**	0.680	0.699

**TABLE IV:** K-Space Interpolation Errors for Different *N*_block_’s With *K* = 5

	NMSE			
	*N*_block_ = 1	*N*_block_ = 3	*N*_block_ = 5	*N*_block_ = 7

*R* = 2	0.384	**0.377**	**0.377**	0.383
*R* = 3	0.531	**0.500**	0.505	0.513
*R* = 4	0.705	0.663	**0.660**	0.682

**TABLE V:** Performance in Tissue Quantification for the GCN and the Cartesian CNN for 1 mm Resolution and *R* = 6

		Relative-Ll	SSIM	PSNR

T1	GCN	**0.126**	**0.953**	**22.74**
	Cartesian CNN	0.130	0.950	22.61

T2	GCN	**0.151**	**0.931**	**23.71**
	Cartesian CNN	0.154	0.927	23.54

## References

[1] Mehta BB , “Magnetic resonance fingerprinting: A technical review,” Magn. Reson. Med, vol. 81, no. 1, pp. 25–46, 2019. 30277265 10.1002/mrm.27403

[2] Majumdar S, Orphanoudakis SC, Gmitro A, O’Donnell M, and Gore JC, “Errors in the measurements of T2 using multiple-echo MRI techniques. I. Effects of radiofrequency pulse imperfections,” Magn. Reson. Med, vol. 3, no. 3, pp. 397–417, Jun. 1986. 3724419 10.1002/mrm.1910030305

[3] Stikov N, Boudreau M, Levesque IR, Tardif CL, Barral JK, and Pike GB, “On the accuracy of t1mapping: Searching for common ground,” Magn. Reson. Med, vol. 73, no. 2, pp. 514–522, Feb. 2015. 24578189 10.1002/mrm.25135

[4] Christodoulou AG , “Magnetic resonance multitasking for motion-resolved quantitative cardiovascular imaging,” Nature Biomed. Eng, vol. 2, no. 4, pp. 215–226, Apr. 2018. 30237910 10.1038/s41551-018-0217-y PMC6141200

[5] Wang F , “Echo planar time-resolved imaging (EPTI),” Magn. Reson. Med, vol. 81, no. 6, pp. 3599–3615, Jun. 2019. 30714198 10.1002/mrm.27673 PMC6435385

[6] Ma D , “Magnetic resonance fingerprinting,” Nature, vol. 495, no. 7440, pp. 187–192, Mar. 2013. 23486058 10.1038/nature11971 PMC3602925

[7] Badve C , “MR fingerprinting of adult brain tumors: Initial experience,” Amer. J. Neuroradiol, vol. 38, no. 3, pp. 492–499, 2017. 28034994 10.3174/ajnr.A5035 PMC5352493

[8] Chen Y , “Three-dimensional MR fingerprinting for quantitative breast imaging,” Radiology, vol. 290, no. 1, pp. 33–40, Jan. 2019. 30375925 10.1148/radiol.2018180836 PMC6312432

[9] Yu AC , “Development of a combined MR fingerprinting and diffusion examination for prostate cancer,” Radiology, vol. 283, no. 3, pp. 729–738, Jun. 2017. 28187264 10.1148/radiol.2017161599 PMC5452885

[10] Ma D , “Fast 3D magnetic resonance fingerprinting for a whole-brain coverage,” Magn. Reson. Med, vol. 79, no. 4, pp. 2190–2197, Apr. 2018. 28833436 10.1002/mrm.26886 PMC5868964

[11] Liao C , “3D MR fingerprinting with accelerated stack-of-spirals and hybrid sliding-window and GRAPPA reconstruction,” NeuroImage, vol. 162, pp. 13–22, Nov. 2017. 28842384 10.1016/j.neuroimage.2017.08.030 PMC6031129

[12] Chen Y, Fang Z, Hung S-C, Chang W-T, Shen D, and Lin W, “High-resolution 3D MR fingerprinting using parallel imaging and deep learning,” NeuroImage, vol. 206, Feb. 2020, Art. no. 116329. 10.1016/j.neuroimage.2019.116329 PMC7136033 31689536

[13] Lee D, Yoo J, Tak S, and Ye J, “Deep residual learning for accelerated MRI using magnitude and phase networks,” IEEE Trans. Biomed. Eng, vol. 65, no. 9, pp. 1985–1995, Sep. 2018. 29993390 10.1109/TBME.2018.2821699

[14] Eo T, Jun Y, Kim T, Jang J, Lee H-J, and Hwang D, “KIKI-net: Cross-domain convolutional neural networks for reconstructing undersampled magnetic resonance images,” Magn. Reson. Med, vol. 80, no. 5, pp. 2188–2201, 2018. 29624729 10.1002/mrm.27201

[15] Akçakaya M, Moeller S, Weingärtner S, and Uğurbil K, “Scan-specific robust artificial-neural-networks for k-space interpolation (RAKI) reconstruction: Database-free deep learning for fast imaging,” Magn. Reson. Med, vol. 81, no. 1, pp. 439–453, Jun. 2019. 30277269 10.1002/mrm.27420 PMC6258345

[16] Hamilton JI, Currey D, Rajagopalan S, and Seiberlich N, “Deep learning reconstruction for cardiac magnetic resonance fingerprinting T_1_ and T_2_ mapping,” Magn. Reson. Med, vol. 85, no. 4, pp. 2127–2135, 2021. 33107162 10.1002/mrm.28568 PMC10250104

[17] Fang Z , “Deep learning for fast and spatially constrained tissue quantification from highly accelerated data in magnetic resonance fingerprinting,” IEEE Trans. Med. Imag, vol. 38, no. 10, pp. 2364–2374, Oct. 2019. 10.1109/TMI.2019.2899328 PMC6692257 30762540

[18] Liu Y, Chen Y, and Yap P-T, “Real-time mapping of tissue properties for magnetic resonance fingerprinting,” in Proc. Int. Conf. Med. Image Comput. Comput.-Assist. Intervent. Cham, Switzerland: Springer, 2021, pp. 161–170. 10.1007/978-3-030-87231-1_16 PMC10496008 37701914

[19] Cohen O, Zhu B, and Rosen MS, “MR fingerprinting deep reconstruction network (DRONE),” Magn. Reson. Med, vol. 80, no. 3, pp. 885–894, Sep. 2018. 29624736 10.1002/mrm.27198 PMC5980718

[20] Fan H, Su P, Huang J, Liu P, and Lu H, “Multi-band MR fingerprinting (MRF) ASL imaging using artificial-neural-network trained with high-fidelity experimental data,” Magn. Reson. Med, vol. 85, no. 4, pp. 1974–1985, Apr. 2021. 33107100 10.1002/mrm.28560

[21] Chen D, Davies ME, and Golbabaee M, “Compressive mr fingerprinting reconstruction with neural proximal gradient iterations,” in Proc. Int. Conf. Med. Image Comput. Comput.-Assist. Intervent. Cham, Switzerland: Springer, 2020, pp. 13–22.

[22] Perlman O , “Quantitative imaging of apoptosis following oncolytic virotherapy by magnetic resonance fingerprinting aided by deep learning,” Nature Biomed. Eng, vol. 6, no. 5, pp. 648–657, May 2022. 34764440 10.1038/s41551-021-00809-7 PMC9091056

[23] Cheng F, Chen Y, Zong X, Lin W, Shen D, and Yap P-T, “Acceleration of high-resolution 3D MR fingerprinting via a graph convolutional network,” in Proc. Int. Conf. Med. Image Comput. Comput.-Assist. Intervent., vol. 12262, 2020, pp. 158–166. 10.1007/978-3-030-59713-9_16 PMC10950303 38504822

[24] Hamilton JI , “MR fingerprinting for rapid quantification of myocardial T_1_, T_2_, and proton spin density,” Magn. Reson. Med, vol. 77, no. 4, pp. 1446–1458, Apr. 2017. 27038043 10.1002/mrm.26216 PMC5045735

[25] Seiberlich N, Lee G, Ehses P, Duerk JL, Gilkeson R, and Griswold M, “Improved temporal resolution in cardiac imaging using through-time spiral GRAPPA,” Magn. Reson. Med, vol. 66, no. 6, pp. 1682–1688, Dec. 2011. 21523823 10.1002/mrm.22952 PMC3247784

[26] He K, Zhang X, Ren S, and Sun J, “Deep residual learning for image recognition,” in Proc. IEEE Conf. Comput. Vis. Pattern Recognit. (CVPR), Jun. 2016, pp. 770–778.

[27] Kipf TN and Welling M, “Semi-supervised classification with graph convolutional networks,” 2016, arXiv:1609.02907.

[28] Fang Z, Chen Y, Nie D, Lin W, and Shen D, “RCA-U-Net: Residual channel attention U-Net for fast tissue quantification in magnetic resonance fingerprinting,” in Proc. Int. Conf. Med. Image Comput. Comput.-Assist. Intervent. Cham, Switzerland: Springer, 2019, pp. 101–109. 10.1007/978-3-030-32248-9_12 PMC7065675 32161930

[29] Song P, Eldar YC, Mazor G, and Rodrigues MRD, “Magnetic resonance fingerprinting using a residual convolutional neural network,” in Proc. IEEE Int. Conf. Acoust., Speech Signal Process. (ICASSP), May 2019, pp. 1040–1044.

[30] Golbabaee M, Chen D, Gomez PA, Menzel MI, and Davies ME, “Geometry of deep learning for magnetic resonance fingerprinting,” in Proc. IEEE Int. Conf. Acoust., Speech Signal Process. (ICASSP), May 2019, pp. 7825–7829.

